# Strategies for Healthy Eating Promotion and Behavioral Change Perceived as Effective by Nutrition Professionals: A Mixed-Methods Study

**DOI:** 10.3389/fnut.2020.00114

**Published:** 2020-08-14

**Authors:** Josiemer Mattei, Charmaine Alfonso

**Affiliations:** ^1^Department of Nutrition, Harvard TH Chan School of Public Health, Boston, MA, United States; ^2^College of Nutritionists and Dietitians of Puerto Rico, San Juan, PR, United States; ^3^School of Health Sciences, Ana G. Méndez University, Gurabo Campus, Gurabo, PR, United States

**Keywords:** nutrition professional, healthy eating promotion, nutrition counseling, Puerto Rico, mixed methods research

## Abstract

Nutrition professionals may recognize ways to improve diet among their clients/patients. This study aimed to survey strategies and foods that nutrition professionals in Puerto Rico perceive as most effective for healthy eating promotion and behavioral change. The study was a cross-sectional online mixed-methods survey conducted among registered members of the College of Nutritionists and Dietitians of Puerto Rico. Using close-ended questions, nutrition professionals identified foods that they considered as easy to include or difficult to control in the diet of their clients/patients, and strategies that may work best for healthy eating. Frequencies of responses were analyzed. Open-ended questions were qualitatively analyzed in NVivo v11. The response rate was 33.2% (*n* = 414). The foods deemed as easy to include in the diet were root vegetables (66%), fruit (66%), legumes (57%), water (38%), and yogurt/dairy (37%). The foods deemed as more difficult to control were sugary beverages (63%), sweets and desserts (57%), fats and fried foods (56%), salt (50%), and white rice (44%). The strategies for healthy eating deemed effective were personalized orientation (79%), setting short-term goals (61%), making gradual dietary changes (53%), and setting health-oriented (41%), and personal (37%) goals. Emerging themes from qualitative analysis included the intuited key role of nutrition professionals, the need for policy changes, emphasizing prevention, cultural sensitivity, and practical issues. Respondents suggested potential strategies across levels of the socioecological model. In conclusion, healthy eating strategies and foods perceived by nutrition professionals as effective may shape optimal nutritional counseling and population-wide approaches to improve healthy eating in Puerto Rico.

## Introduction

Adhering to a healthy diet rich in plant-based foods and low in processed energy-dense foods can help prevent and control multiple chronic diseases such as type 2 diabetes, hypertension, heart disease, and some types of cancer (1). Thus, efforts to encourage healthy lifestyles are made at the individual level by counseling patients toward behavioral change, as well as at the community and population levels through educational, environmental, and policy approaches. Despite these efforts, following a healthy diet continues to be challenging, as evidenced by the low diet quality reported for most residents of the United States, with well-documented disparities for low socioeconomic and racial/ethnic minority groups (2–4). Specifically, the population of Puerto Rico (PR) has inequitable health-related behaviors, including low diet quality and poor nutritional intake (5–10), as well as the high prevalence of multiple chronic diseases that may be prevented by following a healthy lifestyle (11). It is thus important to understand which strategies for healthy eating may be appropriate for a population, especially underserved individuals with inequitable health.

Many formative studies have been conducted to understand the drivers and challenges for individuals and community members to adhere to healthy lifestyles toward disease prevention. Yet, health providers may offer different perspectives on the health beliefs, values, and practices of their patients and their community (12). In particular, nutrition professionals (i.e., nutritionists, dietitians, educators, and others in the field) may have insights on the barriers and motivators for healthy eating of their patients or consumers and in their role in guiding lifestyle change (13, 14), and they may provide care that responds to their patients' cultural norms, values, belief systems, and behaviors (15, 16). Thus, nutrition professionals may provide an additional distinct perspective on which strategies for healthy eating promotion and behavioral change they consider effective in improving diet among their patients and the population they serve. Reports on the perceptions from nutrition professionals are limited, with existing literature focusing on patients with specific conditions (17–19).

In PR, 62% of adults reported obtaining health and nutrition information from a non-physician healthcare professional, such as a nutritionist or dietitian, and 78% of those reported trusting this information (11). Thus, residents of PR may trust and benefit from individual and population-based strategies to improve healthy eating recognized by nutrition professionals. The objective of this mixed-methods survey was to identify the strategies and foods that nutrition professionals in PR perceive as most effective for healthy eating promotion and behavioral change.

## Materials and Methods

### Survey Methodology

The study was designed as a descriptive, self-administered, mixed-methods study (concurrent triangulation design) in a purposive sample of professional members of the College of Nutritionists of Dietitians of Puerto Rico (CNDPR). The strategy and sample source were selected in order to reach most nutrition professionals in PR, as practicing nutritionists/dietitians must be currently licensed (i.e., certified by the PR Review Board), in good standing, and a CNDPR member. CNDPR membership requires a minimum of a bachelor's education with a nutrition degree (e.g., RD, LND), passing the local review board (i.e., license), and completing a coordinated internship program in nutrition/dietetics.

At the time of the survey, the CNDPR had a listserv of 1,271 registered members with listed electronic mail (email) accounts. As the primary sampling unit, members of this listserv were invited to participate in the survey through an email sent by the CNDPR with the subject line “*Survey on strategies for better nutrition in Puerto Rico*.” The subject line had an objective description of the study goals, avoiding language that may influence response rate. The email included an explanation of the study goals and methods, brief assent statement, and a link to the survey. The survey was obtained using the electronic data capture tool Research Electronic Data Capture (REDCap) (20), hosted by Harvard TH Chan School of Public Health and made available for 2 months from August 8, 2016, to October 8, 2016. Only one reminder was sent by email after the first month. CNDPR informed its members of the study on its social media websites. While the system was unable to track the Internet Protocol addresses of responders, the survey instructed participants to submit only one entry and click “submit” only once to avoid repeated entries. In addition, paper-based surveys with the same content and methodology as the online survey were made available at the national CNDPR meeting in August 2016 by having a promotion table at the registration site, where members were informed of the study. Members who approached the table were asked if they had completed the online survey and, if not, were invited to fill the paper-based survey. At that time, CNDPR had no concurrent surveys or studies that would have been confused with this one. Data from paper-based surveys were entered *verbatim* and merged with the online entries before analysis.

The survey was available in Spanish and may be completed in ~10 min. No personal or sociodemographic information was collected. The study did not collect characteristics of non-responders. Individuals consented by participating in the survey. The study was deemed exempt from the Institutional Review Board by Harvard TH Chan School of Public Health on July 2016.

### Question Development and Content

The authors drafted six structured questions: (1) practice type and (2) age and condition of patients mostly served, (3) foods and nutrients deemed as difficult to control, and (4) foods and nutrients deemed as easy and acceptable to eat healthy; and strategies for healthy eating that they perceived as (5) most effective or (6) ineffective. All questions were closed-ended with specific choices given to participants (all possible response options are shown in the Results section). However, participants had the opportunity to enter other options at the end in a blank space. The closed-ended choices were informed by formative research conducted by the team among Puerto Ricans; published studies (21–24); and advice from expert nutrition professionals in community and public health nutrition, clinical nutrition, and nutrition education. For the concurrent qualitative analysis, a final open-ended question was included to openly probe comments (i.e., open text) regarding the status of nutrition in PR and how to improve it, with the goal of capturing other themes or strategies not mentioned in the closed-ended questions as well as to contextualize quantitative responses. The open-ended question was “*Do you have any other comments about nutrition in PR and how to improve it?*” and had unlimited space for a response. The questions were pilot-tested among six Spanish-speaking native Puerto Rican nutrition professionals with diverse positions and expertise (including one community nutritionist, two nutrition educators, and three practicing licensed dietitians) before administrating the survey, to confirm clarity and suitability to the population. Changes made based on the piloting included adding the option to enter “other” in the closed-ended questions, reclassifying the options for practice type, and using additional common names for some foods and beverages.

### Statistical and Qualitative Analysis

Of the 1,271 email addresses in the CNDPR listserv, 24 were returned as inactive or incorrect. From the 1,247 registered members with active emails, 391 online responses were received. One survey was a blank entry, and 10 surveys were excluded because they were duplicates. Thus, there were 380 online responses for a 30.5% response rate of the online survey. In addition, 34 paper-based surveys were filled at the national CNDPR meeting. Thus, the final sample size included 414 participants, for a 33.2% response rate.

Frequencies of responses for all respondents were calculated using SAS v9.4 (SAS Institute, Inc). For qualitative analysis, the responses provided in the final open-ended question were transcribed *verbatim* into a separate text file by a research assistant. Content analysis of this text file was conducted on NVivo software version 11 (QSR International Pty Ltd., 2012) in the original language that it was transcribed (i.e., predominantly Spanish with some English terms) by two Puerto Rican bilingual trained researchers, first independently and then jointly. With a grounded theory inductive approach, the participants' responses were placed into nodes based on resulting main topics, and then meaningful units from each node were used to develop a coding scheme of key themes. The coding scheme was modified as new themes emerged until a final coding scheme of themes was developed. Quotes related to each theme were segregated and sorted to determine the most common responses. Quotes that best represented each theme were selected and translated into English. Concurrent methodological data triangulation was done to detect convergence, complementarity, or discrepancy. Upon cross-examination, there was consensus between authors in the coding, resulting themes, selected representative quotes, and triangulation of results.

## Results

### Quantitative Results

From the 414 participants, most respondents were nutrition professionals working in private hospitals or clinic (40.8%), followed by professionals in public hospitals/clinics (25.9%), and in private practice (20.1%). Other mentioned roles (23.3%) included positions at the Women, Infants and Children (WIC) program (6.0% of all responses), Head Start program (3.1%), community health positions (2.7%), administrative roles (1.9%), teaching (1.5%), and federal programs (1.5%). Respondents mostly served adult populations between the ages of 18 and 64 y (61.8%), followed by adults older than 65 y (58.7%). Children aged 1–12 y and patients with a specific condition were each served by ~37% of the respondents. The least frequently served age group was adolescents 13–17 y (19.3%). The main conditions among served patients were diabetes (14.7%), renal conditions (11.1%), hypertension (5.6%), cancer (3.4%), overweight/obesity (3.1%), cardiovascular disease (2.7%), dyslipidemia (2.4%), and gastrointestinal conditions (1.7%). Additionally, 3.1% mentioned that they counseled all types of conditions.

**Figure 1:**
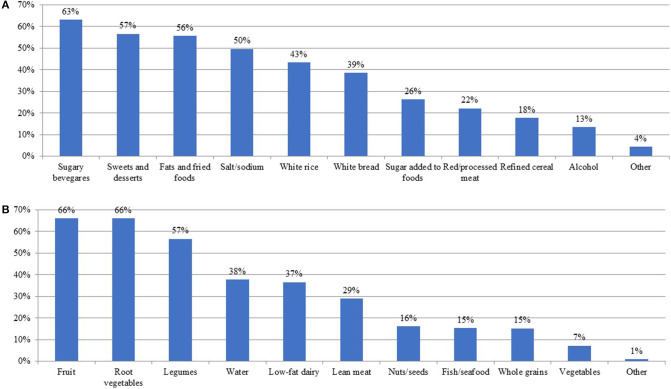
Food or nutrients perceived by nutrition professionals as part of the diet of their patients/clients. Shown as percentages from 414 nutrition professionals surveyed in Puerto Rico that perceive the food or nutrient as difficult to control **(A)** or as easy to include **(B)** in the diet of their patients/clients.

Respondents perceived that foods difficult to control in the diet of their patients were sugary beverages (e.g., sodas, fruit juices, nectars; 63.3%), sweets and desserts (e.g., cakes, pastries, ice cream, cookies; 56.8%), fats and fried foods (55.8%), sodium/salt (49.8%), white rice (43.5%), and white bread (38.7%) (Figure 1A). Other difficult foods mentioned in the blank field included milk (especially whole), fast foods and processed foods, salty snacks, root vegetables, chocolate, seasoning cubes or packages, bakery bread, and proteins (in general). The foods that respondents perceived as easy for their patients to include in their diet were fruits (66.2%), root vegetables or tubers (66.2%), legumes (e.g., beans, chickpeas, lentils; 56.8%), yogurt or healthy dairy products (36.7%), water (37.9%), and lean meats (29.0%) (Figure 1B). Other foods that respondents deemed easy to incorporate in the diet mentioned in the blank field were white rice and sugar-free brownies, snack bars, or candy.

**Figure 2:**
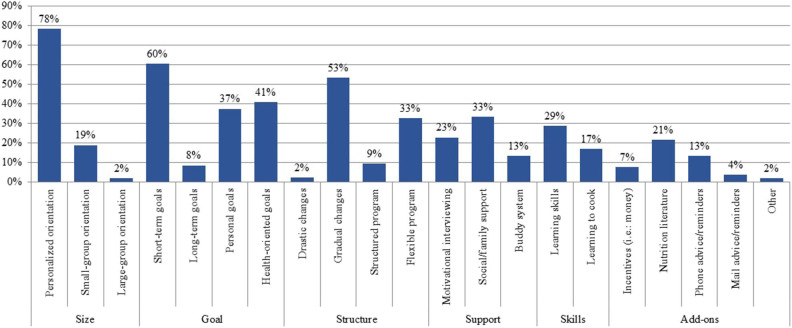
Strategies perceived by nutrition professionals as effective for behavioral change toward healthy eating. Shown as percentage from 414 nutrition professionals surveyed in Puerto Rico that perceive a strategy as effective for behavioral change toward healthy eating. Strategies were combined into general themes: size, goals, structure, support, skills, and add-ons.

The strategies perceived by respondents as effective to support healthy eating included individual and personalized orientation or education (78.5%), setting short-term goals (60.6%), making gradual changes to their diet (53.4%), setting health-oriented (40.8%) or personal goals (37.4%), and having social and family support (33.3%) (Figure 2). Other effective strategies suggested in the blank field included not labeling healthy eating as “diet,” including parents during child education, using visual guides and/or videos at orientation, showing portion size models, and adding social media support. Perceived least effective strategies included orientation or education in large groups (more than 15 people), making drastic changes to the diet, sending tips or cues by mail, material incentives, setting long-term goals, and structured programs. Other strategies that respondents deemed ineffective mentioned in the blank field included the use of meal plans and putting pressure to eat healthy.

### Qualitative Results

Of the 414 responses, 98 participants provided comments in the open-ended question on the nutrition status of PR. Six resulting themes emerged: (1) the intuited key role of nutrition professionals in promoting healthy eating in PR, (2) the need for policy changes, (3) emphasizing primary prevention, (4) the importance of cultural sensitivity, (5) considering practical issues (i.e., cost, skills, education), and (6) reaffirming the predominance of unhealthy foods in PR (Table 1).

**Table 1:** Emerging themes and representative quotes on healthy eating strategies as perceived by nutrition professionals, *n* = 98.

**Emerging theme**	**Representative quotes**
Key role of nutrition professionals	• The nutrition professional should get his/her mind out of the books, diets, and classes. Should focus on the actual reality of the island; be more empathic, charismatic, motivating, and realistic. Should focus on educating people in an interactive way, easy to understand, and leaving behind outdated recommendations. • Nutrition professionals should have the difficult task of reaching more communities and educating our population. We should be more proactive, and focus on primary prevention and giving strategies and tools to each person so that they can adopt nutrition and physical activity as part of their lifestyle, and not do a diet with a deadline. • The health professional should be an example and give the example; there are too many nutritionists who are overweight or obese; is a complaint from patients. • Offer more recognition to the work of the nutritionists as a useful tool to improve health. • Work to eradicate the nutrition clubs and other individuals who have proliferated and are overshadowing the work of the licensed professional in nutrition and dietetics.
Need for policy changes	• If the social macro of the government would give [nutrition] the significance that it deserves, surely we can better educate the community. • If there were more nutritional diagnoses within health insurances, people would look for [nutrition] services more, since the insurance would cover it or would have a minimal fee. • We must work with public policy. For example, in fast food restaurants, substituting soda for water without additional charge was an important achievement. • We should develop public policy to pay additional taxes for fast foods; and to charge an additional fee for choosing sodas or other sugary beverages instead of water. • We should develop a public policy for primary prevention. Access to nutritional orientation is limited in many sectors of the country.
Primary prevention	• Since the patient goes to the clinic for the first time, we should talk to him/her about the importance of prevention and not wait for the person to have a condition or for his/her health to be compromised to want to make very drastic changes to the diet that the patient cannot afford neither financially not emotionally. • The problem of nutrition is that it does not act on prevention; primary care physicians do not refer patients to nutritionists, and specialists refer patients that are already compromised. • There is a need to emphasize the preventive focus and for doctors from all specialties to educate and refer on time. • Nutrition education should start since early ages.
Importance of cultural sensitivity	• Nutrition professionals should return to foods and to the kitchen, breaking away from “single nutrient” recommendations, and implementing nutritional recommendations that take into consideration the cultural tastes, preferences, and influences of the client instead of providing translations of the United States recommendations. • Take into account the cultural background of the Puerto Rican cuisine, showing that it can be healthy and taste good. The importance of respecting the cultural-psychological setting of the patient, validate his/her tastes and preferences. • There is a very common concept that they cannot eat rice and bread. They avoid and blame them as foods that cause their problems. They are basic foods in the Puerto Rican diet. • We should be more flexible with our patients and see their needs and tastes to be able to offer a plan that they want and can follow.
Practical issues (cost, skills, education)	• Teach the mother, father, or caregiver to read the nutrition panel so that they can buy healthier foods. • Include a curriculum on healthy lifestyles in all schools, kindergarten to high school. • People never understand a daily meal plan, don't know how to identify healthy foods. What they need are specific menus and to be accompanied to the supermarket. • Fresh and healthy foods too costly.
Reaffirm predominance of unhealthy foods	• Too much fried stuff and many desserts with sugar. • The big demand for cheap, junk food and fast foods makes this even harder. • There is a poor intake of fruit and vegetables. • Laziness has taken over our youth, they don't cook. They reward good behavior from children with candy, pastries, and sweet desserts. This has led to an addiction to sweets and to think that sweet is better than healthy foods.

Respondents felt that nutrition professionals should have a prominent, proactive, and exemplary role in educating the population on healthy eating using up-to-date recommendations, with empathic and realistic goals that account for the cultural reality of PR. There were also several comments in support of recognizing the nutrition professional as a key player in healthcare, and disapproving unqualified individuals who call themselves nutrition experts. Along this line, a second theme emerged on the need for policy changes, which included getting more support to the nutrition field from the government and from health insurance companies. Also, respondents commented on the need for public policy on food access, taxes on unhealthy foods, and promotion of primary prevention across the island.

Primary prevention emerged strongly as a theme. Respondents stressed its urgency, especially because physicians do not prioritize it. Many respondents mentioned being referred to patients with a diagnosed condition rather than earlier when it could have been prevented. Several comments were also made on addressing prevention at early life stages.

Another theme was the importance of cultural sensitivity when providing counseling or population-wide strategies for healthy eating. Many respondents suggested that nutrition professionals should consider taste and culinary preferences in PR, even recognizing “unhealthy” foods that are staples in the diet of PR and should be decreased but not eliminated. Comments were made on using culturally appropriate materials (avoiding the usual translated materials from the US that may not relate to PR) and being culturally understanding. Relating to this, a theme emerged on the practical issues that would hinder healthy eating among PR residents, especially low nutrition knowledge and skills (education was suggested in early childhood and as a formal school course) and the high cost of healthy foods. A final theme emerged reaffirming the current access to and intake of unhealthy foods in PR, with many respondents commenting on the predominance of fried foods, fast foods, junk food, desserts, and sweets and candy, with low intake of fruit and vegetables.

## Discussion

Triangulating the results of this mixed-methods study, nutrition professionals in PR identified ubiquitous unhealthy foods that are difficult to control in the diet of their patients, as well as foods that may facilitate culturally appropriate healthy eating. Triangulated results also indicated strategies that nutrition professionals deem potentially successful for healthy eating, including the need for tailored orientation by a nutrition professional with cultural competency, focus on primary prevention, and supporting strategies through education and policy changes. The respondents' perceptions largely align with current population-based dietary recommendations and evidence-based strategies for healthy eating (25, 26).

This mixed-methods study showed agreement between quantitative and qualitative components regarding strategies that may best improve healthy eating in PR. For example, quantitative results showed that most respondents perceived personalized nutritional counseling as a successful strategy, while qualitative results generated a theme on the importance of the nutrition professional—which may reflect a biased perception from nutrition professionals—both highlighting the need for guided professional behavioral management. Also, setting health-oriented and personal goals was ranked highly, and nutrition professionals supported this in the qualitative comments by mentioning the need to emphasize primary prevention and to adjust the advice to the patient's priorities. Finally, at the population level, social and family support were perceived as a successful strategy, agreeing with the themes on environmental and policy changes that promote healthy eating through community and family activities, such as education and breastfeeding support.

The foods that were mentioned as difficult to eliminate from the diet have all been reported as frequently consumed in PR, especially sugary beverages, sweets and desserts, fats and fried foods, and white rice (6, 7, 9, 10). Other populations have also expressed a high preference for white rice, a staple food in the Latino culture, over potential substitutes such as brown rice, despite a willingness to reduce its intake (27, 28). Intervention studies that have focused on reducing sugary beverages or fat intake have achieved no or very modest reductions (29–31), supporting the difficulty in controlling these foods. Potential healthy food options mentioned in this study included fruit, root vegetables, legumes, and low-fat dairy. As a staple food, legumes are already consumed in PR—although not at recommended amounts—and adults in PR have expressed having positive perceptions and high motivations for the intake of legumes (32). Similarly, many varieties of fruit and root vegetables are locally produced, and health professionals suggested strengthening local agricultural policies, which could facilitate the access and intake of these foods. Interventions promoting dairy foods may also modestly impact intake (33). In general, the foods listed by respondents reflect the population-based recommendations in the dietary guidelines for Puerto Ricans (25).

Sustaining healthy behaviors is difficult, and respondents in this study considered that guided, individualized counseling and support from a nutritional professional may facilitate the process. In a qualitative study, dietitians in Israel similarly suggested that most patients preferred individualized, not standardized, treatment (13). The authors concluded that dietitians should adopt a more therapeutic approach that relates to patients' cultural needs and desires, in order to achieve sustainable changes in patients' eating patterns. Canadian dietitians also reported that practicing in a client-centered manner is important, yet they noted challenges in balancing their practice values and beliefs with the realities of their work environments (34). Although not explicitly mentioned, comments by the respondents regarding the lack of support to the nutrition field from the healthcare and government sectors and the persistent availability of unhealthy foods may imply that nutrition professionals in PR face similar challenges.

Nutritional messages should additionally be tailored to the cultural needs and preferences of the population, according to the respondents of the current study. Cultural competency in the nutrition field has been strongly advocated (35, 36), and culturally enhanced interventions have been deemed as more effective in improving health outcomes than usual care or other controlled conditions (37). In the current study, nutrition professionals suggested allowing traditional foods important to the culture, such as white rice, even if not as healthy. A recent review concluded that both permissive and restrictive nutrition messages may effectively modify behavior to reduce the intake of discretionary food choices, mainly among younger age groups, with strategies such as restricting portion size, substituting food choices, and supplementing the diet with nutrient-dense foods (38). In addition, both surface and deep cultural considerations should be included when delivering nutritional messages to diverse populations, that is, from recognition of basic external characteristics (such as language) to the core cultural values that may influence specific health behaviors (39, 40). This further validates the theme on the role of the nutrition professional as someone who is likely to understand to the core the eating behaviors and idiosyncrasies of the target population.

Current evidence supports that population-level environmental and system changes through policy as suggested by respondents may be effective in promoting healthy behaviors. A systematic review and meta-analysis concluded that changing the dietary environment may have a larger effect on reducing hemoglobin A1c in adults with type 2 diabetes than changing dietary behavior (41). Evidence-based environmental and policy interventions and programs that may effectively improve nutrition include population education, point-of-purchase labeling, fiscal incentives and disincentives, food assistance programs, procurement of nutrition standards, industry quality standards, school and worksite wellness, food marketing standards, and positive local built environment (26). The need for policy changes was a strong theme emerging in this study. Despite this comment, it has been reported that nutrition educators report being mostly limited to providing information and recommendations on healthy eating rather than being intensively engaged in developing, implementing, and evaluating plans to change environments (42). Thus, involving and training nutrition professionals in policy making and wider community approaches may be needed during professional training.

In agreement with the recommended environmental and policy changes, the respondents considered that there are multiple practical issues that hinder healthy eating among PR residents, including inadequate nutrition knowledge and skills, the high cost of healthy foods, and the predominance of unhealthy foods in the built environment. Education, cost, and access are frequently named challenges to healthy eating across populations (43–46). However, studies have proposed that consuming a health-promoting diet is not necessarily more expensive than unhealthy diet patterns, suggesting that cost may be a perceived rather than an actual barrier to healthy eating (47, 48). Changes to the built environment, such as farmers markets, supermarket availability, and menu labeling, have had a generally positive effect on healthy lifestyle behaviors (49, 50). Thus, it may be possible to overcome the practical barriers mentioned by nutrition professionals through appropriate environmental and policy changes.

In PR, the policy regarding nutrition has been limited, providing an opportunity to enact many policy-oriented suggestions made in this study. Of the few existing policies, PR has food assistance programs (i.e., the Supplemental Assistance Nutrition Program and the Women, Infant, and Children Program), Law Number 256 (year 2015) that mandates all food establishments to allow customers to exchange any beverage for bottled or filtered water (51), and Law Number 427 (year 2000) that allows working nursing mothers a break of up to 1 hour per day for nursing or milk expression for up to a period of 12 months (52). While the main government-sponsored health insurance in PR covers some preventive nutritional services, nutrition professionals in this study strongly suggested strengthening the support to nutrition from both the medical and healthcare fields as well as emphasizing primary, rather than secondary, prevention. The role of doctors in elevating nutrition was mentioned, and having doctors support and advocate for evidenced-based nutrition practice has been deemed as crucial to achieving the World Health Organization's Decade of Action on Nutrition (53). Primary prevention of chronic disease through a healthy diet may help reduce morbidity in PR, as adults in PR with a self-rated fair/poor diet (vs. excellent/good) had twice the odds of having two or more chronic diseases (vs. > 2) (11).

The results of this study represent the subjective views of nutrition providers from PR without comparison to patients' perceptions or outcome-based measures, limiting applicability of results. Similarly, while we cited the effectiveness of the suggested strategies as based on the literature, their effectiveness has not been tested within the PR context. However, the targeted assessment among nutrition professionals was precisely an aim of the study, and the methods may serve as a model for similar surveys in other regions or populations as formative research on challenges and facilitators to healthy eating. A limitation of the study is that it did not collect sociodemographic or job-related data from participants and thus cannot assess if opinions differ by age range, educational attainment or training, years of practice, volume of patients, frequency of appointments, or practitioner's performance. Additionally, the study did not collect characteristics of non-responders to assess response bias. Although the data capturing system did not track entries by identity of responders, the thorough data cleaning excluded only one blank and 10 duplicate surveys. These limitations should be addressed in future research.

Strengths of this study include partnering with CNDPR, of which the registry of nutrition professionals covers PR completely and thus increased the representation of the population served. The response rates in this study of 30.5% for the online strategy only and 33.2% for overall are comparable or higher than reports for other surveys among health professionals, with no incentive and few reminders provided (54, 55). The novel mixed-methods design and analysis allowed us to ascertain a broader context of nutritional needs and recommendations for PR as opposed to using a single data collection strategy.

In conclusion, this mixed-methods study identified strategies and foods that nutrition professionals perceive as most effective for healthy eating promotion and behavioral change in PR, with consensus between qualitative and quantitative results. Providers recognized the need to improve dietary intake and nutritional status in PR. Triangulated results also suggested potential strategies for healthy eating promotion and behavioral change—most with evidence of effectiveness in the literature—that may inform healthy eating recommendations across the Social-Ecological Model for Food and Physical Activity Decisions of the USDA Dietary Guidelines 2020 (Table 2). The model can help health professionals and stakeholders prioritize strategies at all layers of influence that shape a person's food choices and ultimate health outcomes (56). For example, potential strategies at the individual level to improve healthy eating in PR could include encouraging skill building, knowledge, and readiness to change. Potential strategies at environmental settings include supporting programs on breastfeeding, home gardens, and farmers markets and formal nutrition education in schools and communities. Strategies at systems and business sections include policy changes (such as taxes and incentives, healthy school meals, and breastfeeding-promoting policies), emphasizing nutrition in the healthcare system, and improving local agriculture. Finally, fostering a culture of health may help support other levels of the socioecological model by promoting a healthy lifestyle and primary prevention for well-being as part of the culture of PR. Notably, attempted approaches should span all life stages, and socioeconomic and geographical groups.

**Table 2:** Recommendations to improve healthy eating framed within the Social-Ecological Model for Food and Physical Activity Decisions, based on results from a mixed-methods study among nutrition professionals.

**Socioecological model level**	**Recommendations**
**Individual** *Demographics* - Age - Sex - Socioeconomic status - Race/ethnicity - Disability *Other Personal Factors* - Psychosocial - Knowledge and skills - Gene-environment interactions - Food preferences	• Tailor education and programs to age and sex demographics • Consider dietary management within a pre-condition, especially diabetes • Involve parents on nutritional education for children • Build readiness, self-efficacy, and willpower • Instill health awareness, especially family-wide, and intergenerational • Teach healthy food choices, especially for food-assistance programs recipients or low-income individuals • Educate on reading the nutritional facts panel to make choices • Emphasize foods rather than nutrients • Teach healthy cooking skills • Dispel myths and false nutritional information
**Settings** Homes Early care and education Schools Worksites Recreational facilities Food service and retail establishments Other community settings	• Promote home gardens and fresh foods • Extend breastfeeding support programs at various settings • Teach healthy eating in early-childhood programs • Teach a healthy lifestyle curriculum in schools, K-12 • Assign nutritionist/dietitians at strategic sites for food choices (i.e., markets, restaurants) • Push for all food establishments to offer healthy food alternatives • Substitute unhealthy foods with healthy foods at food establishments at no additional cost • Lower costs of healthy foods in food establishments and markets • Galvanize healthy eating community programs, especially in hard-to-reach and poor settings • Create healthy eating programs for adults at-risk of chronic conditions
**Sectors** *Systems* - Government - Education - Health care - Transportation *Organizations* - Public health - Community - Advocacy *Businesses & Industries* - Planning and development - Agriculture - Food and beverage - Manufacturing - Retail - Entertainment - Marketing - Media	• Lower taxes and tariffs on healthy foods • Incentivize healthy foods, especially for food-assistance programs recipients • Tax unhealthy foods (i.e., sugary beverages; fast foods), especially for food-assistance programs recipients • Improve healthfulness of school meals • Increase maternity leave time to promote breastfeeding • Regulate weight loss products • Encourage doctors refer their patients to a nutritionist/dietitian early on • Cover nutritional services by health insurances, especially for prevention • Increase the amount of time for evaluation/counseling of clients/patients • Promote breastfeeding through public health campaigns • Recognize and support nutritionists/dietitians: boost positions, resources, training, education • Regulate that nutritional messages/programs are from qualified experts/sources • Incentivize purchases of local products and produce • Incentivize programs and resources for local agricultural and livestock • Control marketing of junk food • Improve digital technology on nutrition for the Puerto Rico population • Promote healthy eating through mass media
**Social/Cultural Norms** Belief systems Traditions Heritage Religion Priorities Lifestyle Body image	• Support and create a culture of healthy living and prevention • Differentiate dieting vs. healthy eating • Emphasize traditional roots of home cooking as healthy • Consider cultural preferences for flavors, foods, and cooking methods • Keep advice grounded on patients/client's reality and daily life • Convey empathy, motivation, and practicality when advising patients/clients • Connect healthy eating with well-being and health

## Data Availability Statement

The datasets generated for this study are available on request to the corresponding author.

## Ethics Statement

This study was deemed exempt from Institutional Review Board by Harvard TH Chan School of Public Health on July, 2016.

## Author Contributions

JM and CA designed the study, collected, and analyzed the data. JM wrote the manuscript draft with contributions from CA. All authors reviewed and approved the final draft of the manuscript. All authors contributed to the article and approved the submitted version.

## Conflict of Interest

The authors declare that the research was conducted in the absence of any commercial or financial relationships that could be construed as a potential conflict of interest.

## References

[1] Willett WC Koplan JP Nugent R Dusenbury C Puska P Gaziano TA. Prevention of chronic disease by means of diet and lifestyle changes. In Jamison DT Breman JG Measham AR Alleyne G Claeson M. editors. Disease Control Priorities in Developing Countries. Washington, DC; New York, NY: The International Bank for Reconstruction and Development / The World Bank; Oxford University Press. (2006). 21250366

[2] Rehm CD Penalvo JL Afshin A Mozaffarian D. Dietary intake among US adults, 1999-2012. JAMA. (2016) 315:2542–53. 10.1001/jama.2016.7491 27327801 PMC6287627

[3] Thomson JL Tussing-Humphreys LM Goodman MH Landry AS. Diet quality in a nationally representative sample of American children by sociodemographic characteristics. Am J Clin Nutr. (2019) 109:127–38. 10.1093/ajcn/nqy284 30596813

[4] Wang DD Leung CW Li Y Ding EL Chiuve SE Hu FB. Trends in dietary quality among adults in the United States, 1999 through 2010. JAMA Intern Med. (2014) 174:1587–95. 10.1001/jamainternmed.2014.3422 25179639 PMC5924699

[5] Lopez-Cepero A Valencia A Jimenez J Lemon SC Palacios C Rosal MC. Comparison of dietary quality among puerto ricans living in massachusetts and puerto rico. J Immigr Minor Health. (2017) 19:494–8. 10.1007/s10903-016-0480-5 27534857 PMC5315670

[6] Truesdell E Schelske-Santos M Nazario CM Rosario-Rosado RV McCann SE Millen AE. Foods contributing to macronutrient intake of women living in puerto rico reflect both traditional puerto rican and western-type diets. Nutrients. (2018) 10:1242. 10.3390/nu10091242 30200564 PMC6163587

[7] Soltero SM Palacios C Association between dietary patterns and body composition in a group or Puerto Rican obese adults: a pilot study. P R Health Sci J. (2011) 30:22–7. 10.1016/j.nut.2010.02.011 21449494 PMC3449311

[8] Colon-Lopez V Banerjee G Gertz AM Ortiz AP Calo W Finney-Rutten LJ. Behavioral correlates of fruit and vegetable intake in Puerto Rico: results from the health information national trends survey. P R Health Sci J. (2013) 32:194–9. 24397217 PMC4994519

[9] Colon-Ramos U Perez-Cardona CM Monge-Rojas R. Socio-demographic, behavioral, and health correlates of nutrition transition dietary indicators in San Juan, Puerto Rico. Rev Panam Salud Publica. (2013) 34:330–5. 24553760 PMC4129453

[10] Mattei J Tamez M Bigornia SJ Noel SE Xiao RS Rios-Bedoya CF. Dietary intake and its determinants among adults living in the metropolitan area of Puerto Rico. Nutrients. (2019) 11:1598. 10.3390/nu11071598 31337152 PMC6683066

[11] Mattei J Tamez M Rios-Bedoya CF Xiao RS Tucker KL Rodriguez-Orengo JF. Health conditions and lifestyle risk factors of adults living in Puerto Rico: a cross-sectional study. BMC Public Health. (2018) 18:491. 10.1186/s12889-018-5359-z 29650018 PMC5898045

[12] Kennedy BM Rehman M Johnson WD Magee MB Leonard R Katzmarzyk PT. Healthcare providers versus patients' understanding of health beliefs and values. Patient Exp J. (2017) 4:29–37. 10.35680/2372-0247.1237 29308429 PMC5751953

[13] Endevelt R Gesser-Edelsburg A. A qualitative study of adherence to nutritional treatment: perspectives of patients and dietitians. Patient Prefer Adherence. (2014) 8:147–54. 10.2147/PPA.S54799 24523580 PMC3920924

[14] United States Department of Agriculture Best Practices for Creating Nutrition Education Materials. (2019). Available online at: https://www.choosemyplate.gov/best-practices-creating-nutrition-education-materials (accessed June 18, 2020).

[15] Bilyk HT. Role of registered dietitian nutritionists in the research and promotion of native and cultural foods. J Acad Nutr Diet. (2015) 115(5 Suppl):S31–3. 10.1016/j.jand.2015.02.026 25911518

[16] Wynn CL Raj S Tyus F Greer YD Batheja RK Rizwana Z. Barriers to and facilitators of dietetics education among students of diverse backgrounds: results of a survey. J Acad Nutr Diet. (2017) 117:449–68. 10.1016/j.jand.2016.06.010 27492319

[17] Holt DQ Strauss BJ Moore GT. Patients with inflammatory bowel disease and their treating clinicians have different views regarding diet. J Hum Nutr Diet. (2017) 30:66–72. 10.1111/jhn.12400 27412965

[18] Odgers-Jewell K Isenring EA Thomas R Reidlinger DP. Group-based education for patients with type 2 diabetes: a survey of Australian dietitians. Aust J Prim Health. (2017) 23:364–72. 10.1071/PY16156 28566113

[19] Nucci AM Ellsworth K Michalski A Nagel E Wessel J Section APIF. Survey of nutrition management practices in centers for pediatric intestinal rehabilitation. Nutr Clin Pract. (2018) 33:528–38. 10.1177/0884533617719670 28731841

[20] Harris PA Taylor R Thielke R Payne J Gonzalez N Conde JG. Research electronic data capture (REDCap)–a metadata-driven methodology and workflow process for providing translational research informatics support. J Biomed Inform. (2009) 42:377–81. 10.1016/j.jbi.2008.08.010 18929686 PMC2700030

[21] Mattei J Mendez J Falcon LM Tucker KL. Perceptions and motivations to prevent heart disease among Puerto Ricans. Am J Health Behav. (2016) 40:322–31. 10.5993/AJHB.40.3.4 27103411 PMC4897961

[22] Mattei J Sotres-Alvarez D Daviglus ML Gallo LC Gellman M Hu FB. Diet quality and its association with cardiometabolic risk factors vary by hispanic and latino ethnic background in the hispanic community health study/study of latinos. J Nutr. (2016) 146:2035–44. 10.3945/jn.116.231209 27605403 PMC5037869

[23] Siega-Riz AM Sotres-Alvarez D Ayala GX Ginsberg M Himes JH Liu K. Food-group and nutrient-density intakes by hispanic and latino backgrounds in the hispanic community health study/study of latinos. Am J Clin Nutr. (2014) 99:1487–98. 10.3945/ajcn.113.082685 24760972 PMC4021787

[24] Martinez JL Latimer AE Rivers SE Salovey P. Formative research for a community-based message-framing intervention. Am J Health Behav. (2012) 36:335–47. 10.5993/AJHB.36.3.5 22370435 PMC3294377

[25] Palacios C Angleró I. Puerto Rican guidelines on food and diet quality. In Preedy V Hunter LA Patel V editors. Nutrition and Health. New York, NY: Humana Press (2013). p. 213–24. 10.1007/978-1-4614-7315-2_16

[26] Mozaffarian D Angell SY Lang T Rivera JA. Role of government policy in nutrition-barriers to and opportunities for healthier eating. BMJ. (2018) 361:k2426. 10.1136/bmj.k2426 29898890 PMC5997034

[27] Sudha V Spiegelman D Hong B Malik V Jones C Wedick NM. Consumer acceptance and preference study (CAPS) on brown and undermilled Indian rice varieties in Chennai, India. J Am Coll Nutr. (2013) 32:50–7. 10.1080/07315724.2013.767672 24015699 PMC3769789

[28] Monge-Rojas R Mattei J Fuster T Willett W Campos H. Influence of sensory and cultural perceptions of white rice, brown rice and beans by Costa Rican adults in their dietary choices. Appetite. (2014) 81:200–8. 10.1016/j.appet.2014.06.028 24973509

[29] Tobias DK Chen M Manson JE Ludwig DS Willett W Hu FB. Effect of low-fat diet interventions versus other diet interventions on long-term weight change in adults: a systematic review and meta-analysis. Lancet Diabetes Endocrinol. (2015) 3:968–79. 10.1016/S2213-8587(15)00367-8 26527511 PMC4667723

[30] Abdel Rahman A Jomaa L Kahale LA Adair P Pine C. Effectiveness of behavioral interventions to reduce the intake of sugar-sweetened beverages in children and adolescents: a systematic review and meta-analysis. Nutr Rev. (2018) 76:88–107. 10.1093/nutrit/nux061 29281069 PMC5939855

[31] Vargas-Garcia EJ Evans CEL Prestwich A Sykes-Muskett BJ Hooson J Cade JE Interventions to reduce consumption of sugar-sweetened beverages or increase water intake: evidence from a systematic review and meta-analysis. Obes Rev. (2017) 18:1350–63. 10.1111/obr.12580 28721697

[32] Mattei J Campos H Perceptions and behaviors of legume consumption among Puerto Rican Adults. Health Behav Policy Rev. (2014) 1:38–49. 10.14485/HBPR.1.1.5

[33] Rouf AS Grech A Allman-Farinelli M Assessing the efficacy and external validity of interventions promoting calcium or dairy intake in young adults: a systematic review with meta-analysis. Crit Rev Food Sci Nutr. (2018) 58:2600–16. 10.1080/10408398.2017.1336508 28661721

[34] MacLellan D Berenbaum S. Canadian dietitians' understanding of the client-centered approach to nutrition counseling. J Am Diet Assoc. (2007) 107:1414–7. 10.1016/j.jada.2007.05.018 17659911

[35] Fox M Global food practices, cultural competency, and dietetics. J Acad Nutr Diet. (2015) 115:342–8. 10.1016/j.jand.2014.12.019 25721387

[36] Goody CM Drago L Using cultural competence constructs to understand food practices and provide diabetes care and education. Diabetes Spectr. (2009) 22:43–7. 10.2337/diaspect.22.1.43

[37] Barrera M Jr. Castro FG Strycker LA Toobert DJ. Cultural adaptations of behavioral health interventions: a progress report. J Consult Clin Psychol. (2013) 81:196–205. 10.1037/a0027085 22289132 PMC3965302

[38] Grieger JA Wycherley TP Johnson BJ Golley RK. Discrete strategies to reduce intake of discretionary food choices: a scoping review. Int J Behav Nutr Phys Act. (2016) 13:57. 10.1186/s12966-016-0380-z 27151280 PMC4858928

[39] Resnicow K Baranowski T Ahluwalia JS Braithwaite RL. Cultural sensitivity in public health: defined and demystified. Ethn Dis. (1999) 9:10–21. 10355471

[40] Nierkens V Hartman MA Nicolaou M Vissenberg C Beune EJ Hosper K Effectiveness of cultural adaptations of interventions aimed at smoking cessation, diet, and/or physical activity in ethnic minorities. A systematic review. PLoS ONE. (2013) 8:e73373 10.1371/journal.pone.0073373 24116000 PMC3792111

[41] Cradock KA G OL Finucane FM McKay R Quinlan LR Martin Ginis KA. Diet behavior change techniques in type 2 diabetes: a systematic review and meta-analysis. Diabetes Care. (2017) 40:1800–10. 10.2337/dc17-0462 29162585

[42] Lu AH Dickin K Dollahite J. Development and application of a framework to assess community nutritionists' use of environmental strategies to prevent obesity. J Nutr Educ Behav. (2014) 46:475–83. 10.1016/j.jneb.2014.05.014 25087747 PMC4252838

[43] Chavez-Martinez A Cason KL Mayo R Nieto-Montenegro S Williams JE Haley-Zitin V Assessment of predisposing, enabling, and reinforcing factors toward food choices and healthy eating among hispanics in South Carolina. Top Clin Nutr. (2010) 25:47–59. 10.1097/TIN.0b013e3181d109a0

[44] Eikenberry N Smith C. Healthful eating: perceptions, motivations, barriers, and promoters in low-income Minnesota communities. J Am Diet Assoc. (2004) 104:1158–61. 10.1016/j.jada.2004.04.023 15215777

[45] Goh YY Bogart LM Sipple-Asher BK Uyeda K Hawes-Dawson J Olarita-Dhungana J. Using community-based participatory research to identify potential interventions to overcome barriers to adolescents' healthy eating and physical activity. J Behav Med. (2009) 32:491–502. 10.1007/s10865-009-9220-9 19544091 PMC2863037

[46] Seguin R Connor L Nelson M LaCroix A Eldridge G. Understanding barriers and facilitators to healthy eating and active living in rural communities. J Nutr Metab. (2014) 2014:146502. 10.1155/2014/146502 25574386 PMC4276670

[47] de Abreu M Charlton K Probst Y Li N Crino M Wu JHY. Nutrient profiling and food prices: what is the cost of choosing healthier products? J Hum Nutr Diet. (2019) 32:432–42. 10.1111/jhn.12652 30983056

[48] Reidlinger DP Sanders TA Goff LM. How expensive is a cardioprotective diet? Analysis from the CRESSIDA study. Public Health Nutr. (2017) 20:1423–30. 10.1017/S1368980016003529 28095936 PMC10261383

[49] Mayne SL Auchincloss AH Michael YL. Impact of policy and built environment changes on obesity-related outcomes: a systematic review of naturally occurring experiments. Obes Rev. (2015) 16:362–75. 10.1111/obr.12269 25753170 PMC4789114

[50] MacMillan F George ES Feng X Merom D Bennie A Cook A. Do natural experiments of changes in neighborhood built environment impact physical activity and diet? A systematic review. Int J Environ Res Public Health. (2018) 15:217. 10.3390/ijerph15020217 29373567 PMC5858286

[51] LexJuris de Puerto Rico Ley para ordenar a todo establecimiento que se dedique a la venta de comida a que, en sus ofertas, permita, a solicitud del cliente, intercambiar la bebida por agua embotellada o agua filtrada Published 1996-2015. (2019). Gobierno de Puerto Rico Departamento del Trabajo y Recursos Humanos. Available online at: http://www.lexjuris.com/lexlex/Leyes2015/lexl2015256.htm (accessed June 18, 2020).

[52] LexJuris de Puerto Rico Ley para Reglamentar el Período de Lactancia o de Extracción de Leche Materna. (2000). Gobierno de Puerto Rico Departamento del Trabajo y Recursos Humanos. Available online at: http://www.lexjuris.com/lexlex/leyes2000/lex2000427.htm (accessed June 18, 2020).

[53] Adamski M Gibson S Leech M Truby H Are doctors nutritionists? What is the role of doctors in providing nutrition advice? Nutr Bull. (2018) 43:147–52. 10.1111/nbu.12320

[54] Cook DA Wittich CM Daniels WL West CP Harris AM Beebe TJ. Incentive and reminder strategies to improve response rate for internet-based physician surveys: a randomized experiment. J Med Internet Res. (2016) 18:e244. 10.2196/jmir.6318 27637296 PMC5045523

[55] Cunningham CT Quan H Hemmelgarn B Noseworthy T Beck CA Dixon E. Exploring physician specialist response rates to web-based surveys. BMC Med Res Methodol. (2015) 15:32. 10.1186/s12874-015-0016-z 25888346 PMC4404667

[56] Health.gov Dietary Guidelines 2015-2020. Everyone Has a Role in Supporting Healthy Eating Patterns: The Social-Ecological Model. (2019). Available online at: https://health.gov/dietaryguidelines/2015/guidelines/chapter-3/social-ecological-model/ (accessed June 18, 2020).

